# Factors affecting the prevalence of idiopathic scoliosis among children aged 8–15 years in Prishtina, Kosovo

**DOI:** 10.1038/s41598-021-96398-1

**Published:** 2021-08-18

**Authors:** Bernard Tahirbegolli, Rilind Obertinca, Albina Bytyqi, Blerta Kryeziu, Blerte Hyseni, Besarta Taganoviq, Bujar Shabani

**Affiliations:** 1 Heimerer College, Prishtine, Kosovo; 2 National Sports Medicine Center, Prishtine, Kosovo; 3 Department of Nursing and Midwifery, Medicine Faculty, University of Prishtina, Prishtine, Kosovo; 4 Orthopedic and Traumatology Clinic, University and Clinical Center of Kosovo, Kolegji Heimerer, Veranda D4, Hyrja C Dhe D Lagja Kalabri, 10000 Prishtine, Kosovo

**Keywords:** Health care, Medical research, Risk factors

## Abstract

Prospective study, Level of evidence II. The aim of the study was to assess the prevalence of scoliosis among children aged 8–15 years old and to identify the impact of schoolbag weight in developing adolescent idiopathic scoliosis (AIS). AIS is a common disease whose prevalence varies between countries and gender, with an increased rate among females compared to males. Screening children in primary school settings for idiopathic scoliosis (IS) is an important public health issue and is crucial for early detection, prevention of further deformity, and healthy child growth. Our sample was composed of 1619 pupils from the municipality of Prishtina, surveyed from March to April 2019. Measurements were made with a scoliometer on the basis of the Adams test process. Three measurements were taken for each of the participants. Additionally, all the pupils were subjected to bare-foot height and weight measurements with and without school bags. The mean ± standard deviation age of pupils was 11.67 ± 2.00 years old and 49% were females. The prevalence of the angle of trunk rotation (ATR) ≥ 5 ° was 26.1%, females had 1.49 higher odds (95%CI 1.19–1.86) to develop an ATR of ≥ 5° compared to males. The highest rate of ATR of ≥ 5° was seen among the ninth-grade students (31.3%). 56.5% of 4th grade students carry a schoolbag weighing over 12.5% of body weight. Relatively high prevalence of idiopathic scoliosis was found in primary schools in Prishtina. The highest prevalence was found in students attending the ninth grade, while females gender dominated.

## Introduction

Screening children in primary school settings for idiopathic scoliosis (IS) is crucial for early detection, prevention of further deformity, and healthy child growth. Recent reports have shown that the prevalence of IS among primary and middle-school pupils differ, with a rate of 1.02% reported in China, 2.3% in Turkey, 2.93% in Indonesia, and 5.2% in Germany^1–4^. Meanwhile the prevalence of adolescent idiopathic scoliosis (AIS) differs among boys and girls, by body composition and overweight status, family history of scoliosis and ethnicity^2,5–10^.

Early detection of scoliosis is important for better treatment outcomes. High rates of curve progression and early onset of scoliosis are negative predictive parameters for outcomes in idiopathic scoliosis, such as thoracic insufficiency syndrome^11^. Additionally, in adulthood, postural disorders and, in particular, scoliosis have been known to be a significant issue for the health of workers, thereby making it a public health concern^12^.

Screening programs in schools with scoliometer measurements in the Adam forward bend test have been shown to be the most appropriate method to measure the angle of trunk rotation^1–10^. The most important factor was precision when using the scoliometer^13^. According to Pierre et al. scoliometers have a sensitivity of approximately 100% and a specificity of approximately 47% at an angle of trunk rotation (ATR)/angle of trunk inclination (ATI) reading of 5°, while its sensitivity drops to 83%, but specificity rises to 86% at an ATI reading of^13^ 7°.

Moreover, in school age pupils, one of the factors influencing the increase of the angle of trunk rotation is the weight of the schoolbag and how it is carried^14,15^. Dockrell et al. indicated that schoolbag-related discomfort was reported more frequently in the shoulders than in the back^16^.

To the best of our knowledge this is the first scoliosis survey among pupils from primary schools in Kosovo. The aim of this study is to assess the prevalence of scoliosis among primary school pupils and to identify the impact of schoolbag weight in developing scoliosis.

## Materials and methods

In this research, 1619 students of primary and lower secondary schools in the municipality of Prishtina were surveyed in total during the months of March–April 2019.

In order to determine the sample size, the number of students in primary schools in the Prishtina municipality for the school year 2018–2019 was obtained and stratified by the number of students in each grade. Since the prevalence of scoliosis among primary and lower secondary school students in Prishtina, Kosovo is not known, the sample size was calculated on the basis of the total number of students attending classes in grades 3–9 (22,741 students) and CI 95%, with a 3% Margin Error, leading to a sample size of 1020 students.

To determine the level of reliability between the two researchers who measured all the student angles of trunk rotation with the scoliometer (plastic economy model, #12-1099, Baseline®, New York, USA), the first measurement was performed by both researchers on 142 students and the inter-rater correlation coefficient (ICC) was calculated as follows: 0.867 (95% confidence interval (95% CI) 0.815–0905). To assess the intra-rater reliability the first and second researchers conducted the second measurements on 48 pupils, and ICC (1,1) for the first researcher was 0.772 (95% CI 0.595–872), while the second researcher's ICC (1,1) was 0.799 (95%CI 0.643–0.887).

Measurements were made on the basis of the Adam forward bend test process and three measurements were taken for each of the participants. The first measurement was made at the upper thoracic level at an angle of 45° of flexion in front of the trunk, the second measurement at the lower thoracic level at an angle of 60° and the third measurement at an angle of 90° to the lumbar region.

In any of these measurements, a deviation of ≥ 5° of the angle of the trunk rotation in the scoliometer was suspected of idiopathic scoliosis and the telephone number of the parent was obtained. The parents were contacted, and full length spine radiographs were recommended in order to further investigate the Cobb angle. Follow-up by an orthopaedic surgeon was also recommended.

In addition to scoliometer measurements, all students were assessed for bare-foot body length measurements and weight measurements with school bags and without school bags, hence determining the weight of the school bag. The measurement of these parameters was preceded by a questionnaire containing socio-demographic data for students such as year of birth, class, gender, place of residence, distance traveled to school in minutes, positive family history of spine deformity, the way in which school bags are carried, etc. Which was fulfilled by the researcher based on the students' answers (Table 1). The study protocol was approved by the Heimerer College Institutional Review Board (Protocol no: 069/19) and is complied fully with the provisions and guidelines of the Helsinki Declaration regarding research on human participants. Formal study permits were received from the Educational Directorate of the Pristina Municipality, and the authors have also taken the informed consent from a parent and/or legal guardian permission through the Parents Committee of each school for their children to participate in the study.

**Table 1:** Socio-demographic characteristics of study sample.

	n(%) and/or mean ± SD
**Sex**	
Male	824 (50.9)
Female	795 (49.1)
Age (years)	11.67 ± 2.00
**Age based on school grade (years)**	
3rd (n = 204)	8.52 ± 0.59
4th (n = 170)	9.49 ± 0.72
5th (n = 164)	10.35 ± 1.01
6th (n = 342)	11.46 ± 0.61
7th (n = 273)	12.51 ± 0.66
8th (n = 258)	13.52 ± 0.66
9th (n = 208)	14.48 ± 0.56
Urban	1377 (85.2)
Rural	240 (14.8)
Body weight (kg)	48.22 ± 15.30
Body height (cm)	154.28 ± 13.56
BMI (kg/m^2^)	19.83 ± 4.08
**Sports**	
Yes	883 (54.7)
No	732 45.3)
**Family history for spine deformity**	
Yes	255 (15.8)
No	1364 (84.2)
**Schoolbag carrying**	
Single shoulder	39 (2.6)
Backpack	1435 (96.3)
Trolley case	16 (1.1)
Walking from home to school (min)	10.49 ± 7.14
**School bag weight to body weight rate**	
≤ 12.5%	1266 (78.2)
> 12.5%	353 (21.8)
**Angle of trunk rotation**	
< 5°	1197 (73.9)
≥ 5°	422 (26.1)

Frequencies (n) and percentages (%) were used to summarize categorical variables. Continuous variables are summarized with mean ± standard deviation (SD) or median and InterQuartile Rate (IQR). Distribution of normality was evaluated using Shapiro–Wilk and Kolmogorov–Smirnov tests. The chi-square (χ^2^) test and contingency tables were used to compare the frequency of categorical variables. The student t-test (for two groups), the one-way ANOVA (for more than two groups) and post hoc Tukey test were used to analyse continuous variables. Adjusted and unadjusted binary logistic regression analyses were used to identify significant predictors of the angle of trunk rotation ≥ 5°. The outcomes were statistically analysed by using the IBM SPSS version 24 software^17^.

## Results

The gender distribution of students that participated in the study was almost equal (51% male and 49% female), mean ± SD age was 11.67 ± 2.00 years, less than 15% of them live in rural area (Table 1). In the assessment of the Adam forward bend test, more than 1/4 of the students had a trunk rotation angle of 5 or higher degrees.

Over 15% of children claimed that someone in the family had a spine deformity. More than half of the children reported sporting activity at least twice a week. Over 96% of students were found to be carrying a schoolbag. The students said they walk an average of 10 min on foot from home to school, and vice versa. Over 21% of students were found to carry schoolbags weighing more than 12.5% of their body weight (Table 1).

**Figure 1:**
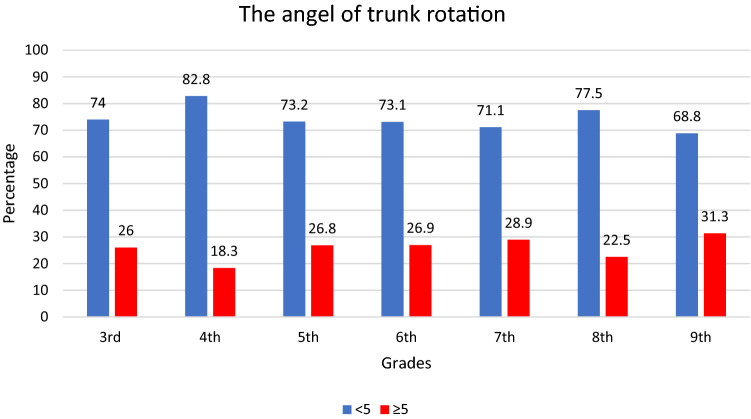
Percentages of children with the angel of trunk rotation < 5° and ≥ 5° according to school grades.

**Figure 2:**
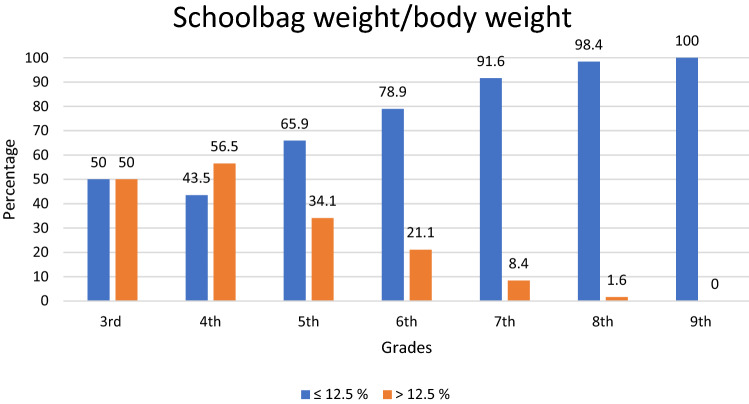
Prevalence of students carrying school bags up to 12.5% and more than 12.5% of body weight.

The highest frequency of those who had an abnormal angle of trunk rotation was seen among students attending the ninth grade (31.3%). Immediately after them there are those in the seventh grade (28.9%), and then those in the sixth grade (26.9%) (Fig. 1). Although, when evaluated separately, 118 (7.3%) of students have a deviation of ≥ 5° of the angle of the trunk rotation measured in 45° forward bend test at the upper thoracic level, 293 (18.1%) in 60° at the lower thoracic level, and 197 (12.2%) in 90° at the lumbar region. Half of students in grade 3 and more than 56% of then in grade 4, and about one-third of the students in grade 5 carry schoolbags weighing more than 12.5% of their body weight (Fig. 2).

In the chi-square test, females were found to have a higher rate of pathological rotation of the trunk angle compared to males (p < 0.05). No statistically significant difference was found in the frequency distribution of the schoolbag weight-to-body weight ratio (up to 10%, 12.5% and 15% and more) in relation to the deviation of the trunk rotation axis below 5° and ≥ 5° (Table 2). 27% (96 out of 353) of pupils with a schoolbag weight-to-body weight ratio greater than 12.5%, and 27% (20 out of 74) of pupils with a schoolbag weight-to-body weight ratio greater than 15% had a trunk rotation axis ≥ 5° (Table 2).

**Table 2:** Frequency distributions of the angle of trunk rotation according to gender and schoolbag weight to body weight rate.

	ATR < 5°	ATR ≥ 5°	
	n(%)	n(%)	
Male	640 (39.5)	184 (11.4)	×2 = 12.150, df = 1, p < .001
Female	557 (34.4)	238 (14.7)	
Schoolbag weight until 10% of BW	843 (52.1)	285 (17.6)	×2 = 1.234, df = 1, p = .267
Schoolbag weight over 10% of BW	354 (21.9)	137 (8.5)	
Schoolbag weight until 12.5% of BW	940 (58.1)	326 (20.1)	X2 = .299, df = 1, p = .584
Schoolbag weight over 12.5% of BW	257 (15.9)	96 (5.9)	
Schoolbag weight until 15% of BW	1143 (70.6)	402 (24.8)	×2 = .037, df = 1, p = .847
Schoolbag weight over 15% of BW	54 (3.3)	20 (1.2)	

ATR, angle of trunk rotation; BW, body weight.

No statistically significant difference was found on schoolbag weight among those with the trunk rotation axis below 5° and ≥ 5° (4.03 ± 1.24 (kg), and 3.93 ± 1.32 (kg), respectively) (p > 0.05). There was also no statistically significant correlation between the percentage of schoolbag weight to body weight and the angle of trunk rotation (p > 0.05).

Only the gender variable was found to be a significant predictor (p < 0.01) in the unadjusted binary logistic regression analysis for the angle of trunk rotation ATR ≥ 5°. Females were 1.49 times more likely than males to develop ATR ≥ 5° (95% CI 1.19–1.86). However, this does not control any other variables.

In the adjusted stepwise model, three variables were significant predictors (p < 0.05), namely gender (Wald = 13.88), body weight (Wald = 7.54), and body height (Wald = 8.52) (Table 3). According to the adjusted model, the risk of ATR ≥ 5° increases by 2% for every cm increase in body height, and by 53% if you are female. However, for each additional kilogram of body weight, the risk of an ATR ≥ 5° decreases by 2%.

**Table 3:** Binary logistic regression model of the predicting wariables of abnormal angle of trunk rotation.

	Unadjusted			Adjusted*		
	Wald	p	ODDS (95% CI)	Wald	p	ODDS (95% CI)
Age	.89	.345	1.03 (.97–1.09)	–	–	–
Female gender	12.08	.001	1.49 (1.19–1.86)	13.88	< .001	1.53 (1.21–1.94)
Body weight	1.27	.260	.99 (.99–1.00)	7.54	.006	.98 (.97–.1.00)
Body height	.86	.355	1.00 (.99–1.01)	8.52	.004	1.02 (1.01–1.04)
Schoolbag weight > 12.5% of BW	0.299	.584	1.08 (.83–1.41)	–	–	–
Transport**	1.34	.248	1.15 (.91–1.47)	–	–	–
Family history***	2.67	.102	.78 (.58–1.05)	–	–	–
Schoolbag carrying****	.48	.490	.78 (.39–1.56)	–	–	–
Constant	–	–	–	14.51	< .001	.031

BW, body weight.

*Here are presented only variables that worked significantly in the model.

**Transportation is a binomial variable consisting of: by walking and by vehicle.

***Family history is a dichotomous variable consisting of: positive history (when someone in the family has spine deviation) and negative history.

****Schoolbag carrying is a dichotomous variable consisting of carrying with one shoulder and carrying in the back.

## Discussion

The main findings of this study are that the prevalence of scoliosis among primary school students in Prishtina was 26.1% and females seems to have higher rates of scoliosis compared to males. The presented results are similar to results reported by Ciaccia et al. (24.3%)^6^, but, significantly higher to those previously presented by Nery et al. (1.4%)^18^. Also, in 2014 Ortega et al. showed a prevalence of 14.2% of scoliosis in Mexican school children with an average age of 10 years^19^. Which is comparable to our prevalence on the lumbar region measurement only (12.2%). The variations in prevalence between studies could be attributed to the different methods used to detect scoliosis as well as the study sample chosen.

According to Yilmaz et al., after screening with a scoliometer, more than 15% were suspected of having AIS based on ≥ 5° ATR, but only 2.3% were confirmed by radiography evaluation^1^. Similarly, Komang-Agung et al. found a higher prevalence of AIS (6.4%) when screening with a scoliometer, but only 2.9% when assessing with a Cobb^2^ angle ≥ 10°.

Our study protocol corresponds to that of Penha et al., who measured ATR at 45°, 60° and 90° trunk flexion^20^. However, Penha et al. used a cut-off of ATR ≥ 7°, whereas we used a cut-off of ATR ≥ 5° for scoliosis^20^.

Yilmaz et al.^1^ had a study sample aged 10–15 years, Nery et al.^18^ had 10–14 years old, Ciaccia et al.^6^ 10–14 years old, and Ortega et al.^19^ had a study sample aged 6–12 years, while in our study participants ranged in age from 8 to 15 years old.

The sample size used in similar articles mentioned above are varying from 954 participants to 2822, which seem not to influence differences between final results on prevalence of scoliosis^6,19^.

We found in the studied population that the highest frequency of those who had an abnormal angle of trunk rotation was seen among students attending the ninth grade (31.3%). These results are in line with a study conducted in Singapore, where the highest prevalence was reported to be in participants at 13–14 years old^21^. Curves typically progress most rapidly during the adolescent growth spurt before skeletal maturity. Furthermore, curves advance in roughly two-thirds of skeletally immature patients before they reach skeletal maturity^22^.

The mean weight of schoolbags carried by students in our study was 4.0 kg which is lighter than that found in Negrini and Carabalona’s study, who measured students with a mean age of 11.7 years and found that their mean schoolbag weight was 9.3 kg^23^. In the present study, it is noticed that 56.5% of 4th grade students carry school bags weighing more than 12.5% of their body weight. Similar results were reported in Ireland^16^.

Findings of the study revealed that females were found to have a higher rate of pathological rotation of the trunk angle compared to males. Previous studies have similarly reported higher prevalence of scoliosis in females^21,24^, while Ciaccia, et al. found no significant difference in the prevalence of scoliosis between genders^6^. The possibility for these variations could be in the age groups studied, as the peak of growth rate in girls occurs prior to boys.

There is no statistically significant difference regarding schoolbag weight in relation to trunk rotation axis. Kouwenhiven and Castelein have shown in their study that there does not exist a single cause for the development of idiopathic scoliosis, thus the condition is termed multifactorial^25^. According to this, schoolbag weight cannot be the sole cause of IS but it can influence IS. In addition, some studies have demonstrated that dorsally directed shear loads act as an enhancer of slight pre-existent vertebral rotation, under critical circumstances during growth^26,27^. Postural sways also were observed when adolescent idiopathic scoliosis subjects carried a backpack loaded with 10% of body weight load^15^.

It is additionally critical to note that the schoolbag carrying method can influence the morphology of the spinal column. Hsu et al. indicate that children who carry backpacks on one shoulder need to compensate for the bag weight by tilting their head position to the opposite side^14^. Furthermore, this strategy creates a high torque around the spine, which may cause scoliosis. However, in our study 96.3% of the participants carried the schoolbags over both shoulders.

Despite the fact that the multifactorial nature of schoolbag-related discomfort in schoolchildren should be considered, school bags weighing more than 10% of BW are associated with a higher occurrence of back pain also^16,28^. Moreover, Sahli et al.^15^ in their study revealed that postural stability and bodily orientation parameters increase with increasing backpack load^15^. Therefore, as indicated by Brackley and Stevenson, and Janakiraman et al. in their studies, a schoolbag load carriage of 10–15% of body weight would be an acceptable weight limit^29,30^.

This study has some limitations First, a small number of participants could be considered a limitation of this study as compared to other large samples in the literature. The size of the sample, however, was calculated on the basis of the total number of students attending classes in grades 3 to 9 (22,741 students) and CI 95%, with a 3% Margin Error leading to a sample of 1020 students. In addition, our study is consistent with other studies about the prevalence and risk factors of scoliosis. The scoliometer measures' reliability and correlation coefficient are, however, a limitation that should be mentioned. Our study's inter- and intra-rater reliability were quite good (ICC > 0.75), which is comparable to the results of scoliometer measurements in the Bonagamba et al. study^31^. Another possible limitation is the lack of radiographic data. Coelho et al. revealed that it is possible to identify 87% of the patients with idiopathic scoliosis lateral curvatures greater than 10° Cobb and 100% of the patients with curves greater than 20° Cobb using 5° as the criteria for referral^32^. The correlation between the scoliometer measurements and radiograph analyses was considered good (r = 0.7, p < 0.05). Furthermore, there are studies which considered appropriate in using scoliometer as a screening device^33,34^.

### Clinical relevance

The most important clinical implication of this study is prevention. Given the high prevalence of scoliosis (26%) from this study, establishing an appropriate screening program would be more than necessary. Early detection of smaller degree-scoliosis has many advantages, including prevention of curve progression, reduced back pain and benefits of conservative treatment^35–37^. Recent publications have announced that a correct conservative treatment can reduce the incidence and prevalence of scoliosis surgery^37–40^.

In conclusion, the relatively high prevalence of idiopathic scoliosis was found in primary school students in Prishtina. The highest prevalence was found in students attending the ninth grade, while IS was more common in female students. On the other hand, carrying schoolbags heavier than 12.5% of body weight does not have any impact on IS. Since, in the current study the highest percentage of the students who carried a schoolbag more than 12.5% of body weight was in 4th grade students but the prevalence of scoliosis dominated in 9th grade, it is recommended that future studies should focus on the prolonged effect of a bag’s weight in spinal curvature. The Ministry of Education and Ministry of Health of Kosovo should work together and put scoliosis investigation within the routine examination of schoolboys and girls in Kosovo.
